# MetHoS: a platform for large-scale processing, storage and analysis of metabolomics data

**DOI:** 10.1186/s12859-022-04793-w

**Published:** 2022-07-08

**Authors:** Konstantinos Tzanakis, Tim W. Nattkemper, Karsten Niehaus, Stefan P. Albaum

**Affiliations:** 1 International Research Training Group “Computational Methods for the Analysis of the Diversity and Dynamics of Genomes”, Faculty of Technology, Bielefeld University, Bielefeld, Germany; 2 Biodata Mining Group, Center for Biotechnology (CeBiTec), Faculty of Technology, Bielefeld University, Bielefeld, Germany; 3 Proteome and Metabolome Research, Center for Biotechnology (CeBiTec), Faculty of Biology, Bielefeld University, Bielefeld, Germany; 4 Bioinformatics Resource Facility, Center for Biotechnology (CeBiTec), Bielefeld University, Bielefeld, Germany

**Keywords:** Mass spectrometry data, Large-scale metabolomics, Parallel processing, Distributed storage, Distributed analysis

## Abstract

**Background:**

Modern mass spectrometry has revolutionized the detection and analysis of metabolites but likewise, let the data skyrocket with repositories for metabolomics data filling up with thousands of datasets. While there are many software tools for the analysis of individual experiments with a few to dozens of chromatograms, we see a demand for a contemporary software solution capable of processing and analyzing hundreds or even thousands of experiments in an integrative manner with standardized workflows.

**Results:**

Here, we introduce MetHoS as an automated web-based software platform for the processing, storage and analysis of great amounts of mass spectrometry-based metabolomics data sets originating from different metabolomics studies. MetHoS is based on Big Data frameworks to enable parallel processing, distributed storage and distributed analysis of even larger data sets across clusters of computers in a highly scalable manner. It has been designed to allow the processing and analysis of any amount of experiments and samples in an integrative manner. In order to demonstrate the capabilities of MetHoS, thousands of experiments were downloaded from the MetaboLights database and used to perform a large-scale processing, storage and statistical analysis in a proof-of-concept study.

**Conclusions:**

MetHoS is suitable for large-scale processing, storage and analysis of metabolomics data aiming at untargeted metabolomic analyses. It is freely available at: https://methos.cebitec.uni-bielefeld.de/. Users interested in analyzing their own data are encouraged to apply for an account.

**Supplementary Information:**

The online version contains supplementary material available at 10.1186/s12859-022-04793-w.

## Background

Metabolomics is a unique part of modern life science and molecular biology due to its multidisciplinary requirements: knowledge from biology, chemistry, physics as well as mathematics and statistics needs to be integrated. It deals with the quantification and identification of small molecules called metabolites (< 1500 Da) which are the intermediate and ending products in cellular processes of an organism [1]. The key technology to investigate the metabolites that are abundant in an organism or a tissue is mass spectrometry, where state-of-the-art methods allow gaining tens of thousands of mass spectra within a few minutes which in turn characterize hundreds of potential compounds. The entirety of these metabolites in a cell at a specific moment can be considered as a high-dimensional molecular snapshot of the organism which carries an imprint of all genetic, epigenetic and environmental factors. Thus, one of the main goals of metabolomics research is to bridge the gap between the genotype and phenotype in order to get a complete picture of the internal structure and behavior of a cell [2]. Metabolomics has a wide application in many different fields such as toxicology assessment, nutritional genomics, biomarker discovery and identification, drug development and disease prognosis [3].

High-throughput metabolomics experiments, generally, follow an untargeted approach which is characterized by the simultaneous measurement of a large number of metabolites from each sample, thus analyzing the global metabolomic profile [4]. In this, raw datasets obtained from compound separation and detection techniques, such as Gas Chromatography (GC) or Liquid Chromatography (LC) coupled to Mass spectrometry (MS), are transformed to quantitative metabolite information [5–7]. The general processing strategy includes noise filtering and baseline reduction [8] followed by peak detection [9] and deconvolution [10], chromatographic alignment [11], identification of metabolomic features [12], substitution of missing values [13], normalization [14] and statistical analysis [15, 16].

To support researchers in the complicated and complex analytical workflow, a large number of software tools have been developed. Some of the available tools are focused either on the quantification or identification of metabolites. For instance, iMet-Q [17] and apLCMS [18] deal with the quantification step while Metabolyzer [19] with the identification of metabolites. Similarly, MetaBox [20] puts the focus only on the statistical analysis that follows the processing step. Other software packages include both the quantification and identification step like XCMS [21], MetAlign 3.0 [22], MZmine 2 [23], MAVEN [24], mzMatch [25] or MS-Dial [26]. However, they either lack statistical capabilities or are not web-based, which confines them at the usage of computational resources. There are also web-based tools like MeltDB [27], XCMS Online [28] and MetaboAnalyst [29] that not only offer support in data storage and retrieval but also analytical tools for quantification, identification and statistical analysis. However, none of these tools is able to deal with large collections of data sets, referred to as large-scale metabolomics. In the context of this work, we categorize data sets ranging from more than a couple of hundreds to thousands of files to be a large-scale metabolomics data set. For instance, MeltDB is a semi-automated system not prepared for big amounts of data and XCMS Online places the focus on processing with restrictions in the storage and limited statistics, while the recently released MetaboAnalyst 5.0 at least supports up to 200 files.

With repositories giving access to hundreds or even thousands of files from different experiments we see a clear demand for an easy-to-use software solution capable of handling large amounts of metabolomics data in short processing and analysis times. In recent years, first big data frameworks and libraries have been developed which have proven to be capable of dealing with such amounts of data. PhenoMeNal [30] is a framework that can handle large volumes of data as it utilizes a cluster of computers. However, it follows the Infrastructure-as-a-Service cloud model and requires prior knowledge of cloud computing concepts, which most metabolomics researchers may not be familiar with. In a similar manner, workflow4metabolomics [31] is a repository for Galaxy-based workflows that covers many aspects of metabolomics data processing and analysis. Yet again this requires set up of the technical infrastructure and does not include a scalable storage solution. In this study, we present MetHoS, a ready-to-use web-based platform for large-scale processing, storage and analysis of metabolomics data sets. MetHoS is based on big data frameworks and provides users efficient and user-friendly handling of their own experimental metabolomics data. With Apache Spark [32], Apache Cassandra [33] and KNIME (Konstanz Information Miner) [34] as our fundament we propose a different way of handling large-scale datasets with the prospect of handling even largest amounts of data.

## Implementation

### Application architecture

**Fig. 1:**
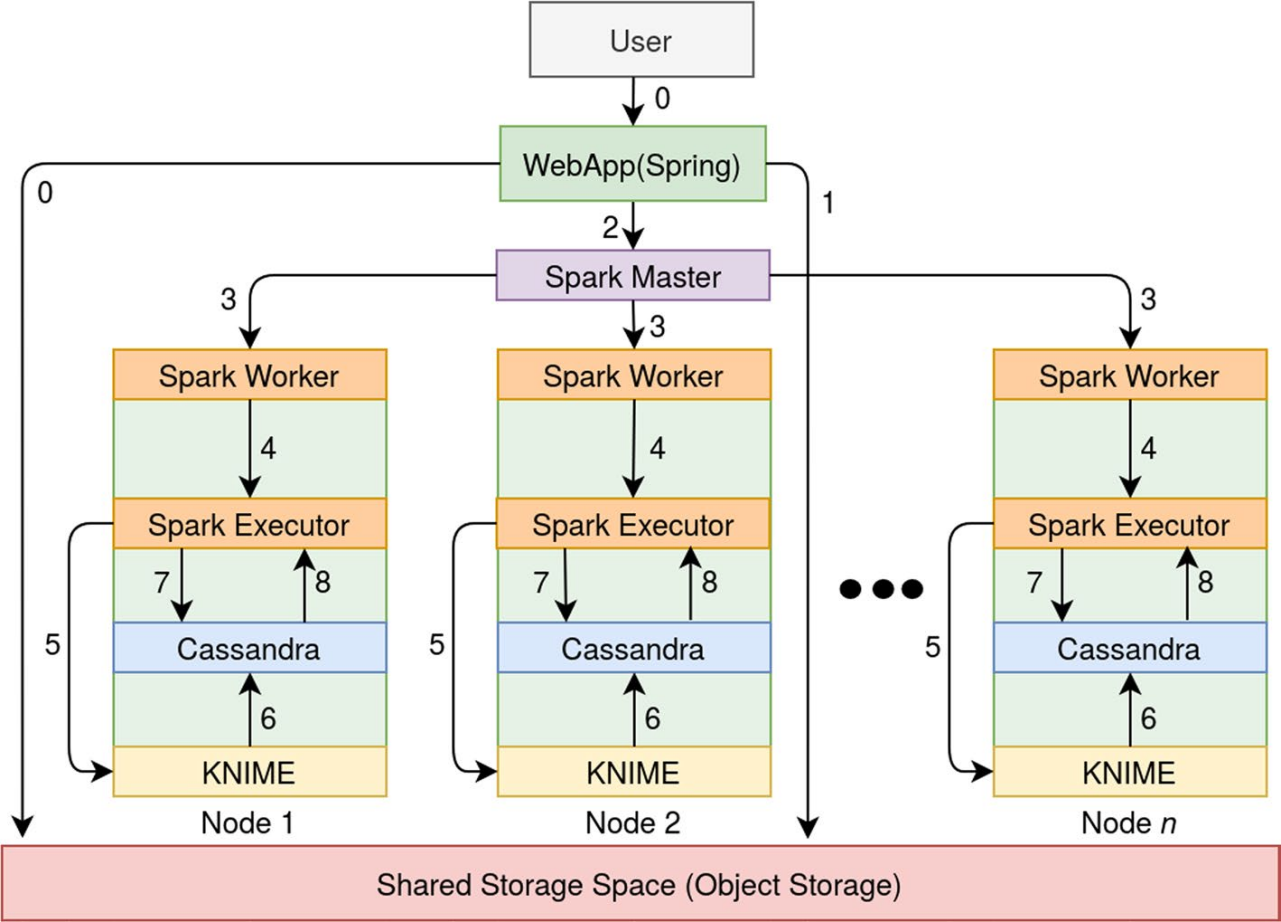
This figure shows the data-flow during the processing and analysis steps of the combination of Apache Spark, Apache Cassandra and KNIME

Cloud computing allows for the parallel execution of tasks on a large number of virtual machines. Moreover, it allows for scalability: if the size of the problem increases, more machines can easily be added. MetHoS supports horizontal scaling, thus ensuring performance by not being limited to the capacity of a single unit and redundancy with no single point of failure. It is written in Java and utilizes a set of software tools that enable parallel processing, distributed storage and distributed analysis on the cloud (Fig. 1). MetHoS uses a variable number of computer nodes and has been designed for OpenStack, the popular open-source software platform for cloud computing.

The general processing of uploaded metabolomics data is relying on the software KNIME, which is a well-known open-source data workflow engine and analytics platform. Apache Cassandra, a NoSQL database for distributed storage, was selected to store the results of the processing across the cluster. We integrated Apache Spark, a fast in-memory cluster-computing engine, that can perform distributed processing and analysis of big amounts of data. Finally, we combined all three software tools together with the Spring Application framework providing users a friendly and easy-to-understand web-based application.

### Project and data management

MetHoS provides sophisticated user and project management, in which uploaded samples can be grouped to create experiments in already pre-defined projects. The owner of a project has the ability to edit it and also manage access rights. MetHoS makes use of OpenStack’s object storage through the swift API, where the experiments are stored in a shared storage space (Fig. 1).

An *experiment* in MetHoS refers to a biological experiment that consists of many biological replicates. After uploading, experiments can be processed (quantification and identification step) by selecting one of the automated KNIME workflows. In result, each workflow is also responsible for storing the results straight into the Cassandra database. Apache Spark verifies fair job-scheduling and workload distribution, e.g. in terms of KNIME workflow processing, giving thus the ability to process thousands of experiments in a matter of hours.

Results of the processing are presented in the *View* section. The representation can be either in a form of a table by selecting an experiment and observing all the identified and unidentified metabolomic features or with boxplots by selecting more experiments and observing the metabolomic features among them. Various statistical tests enable further investigation and analysis of the processed metabolomics data within an experiment but, in particular, across a multitude of experiments. The results are presented and visualized with the help of the D3 Javascript library.

### Parallel processing

The architecture and design of MetHoS are based on flexibility allowing for an easy integration of workflows implemented in KNIME Analytics Platform. KNIME has integrated OpenMS [35], an open-source software C++ library for management and analyses of metabolomics data, allowing for quantification and identification of metabolites, against spectral databases, in each sample or group of samples.

Once the raw chromatographic data are uploaded to the Openstack object storage, they can be selected for processing (Fig. 1, step 1) with a pre-defined workflow. The Spark master, which is a single coordinator that acquires cluster nodes (Spark Workers), receives the request and distributes the tasks to each node (Fig. 1, steps 2 and 3). Then, each Spark Worker activates a Spark Executor, an agent responsible for carrying out the task and activating KNIME on every node in order to process the selected experiments (Fig. 1, steps 4 and 5).

**Fig. 2:**
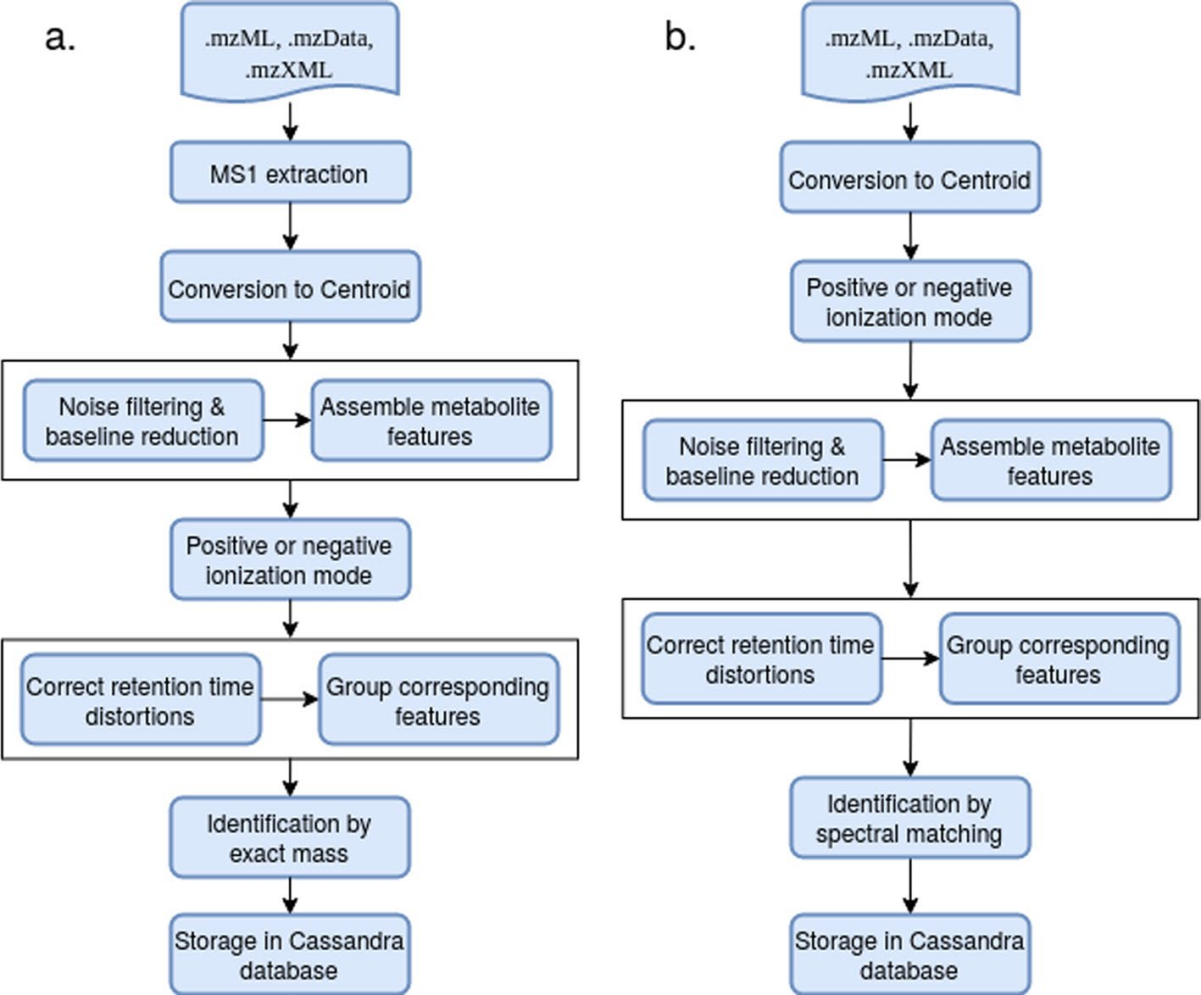
This figure shows the steps of two of the workflows currently implemented in MetHoS. **a** Identification by exact mass, **b** Identification by spectral matching

MetHos supports the well-known and accepted open-source file formats .mzML, .mzData and .mzXML as inputs and produces information about identified and unidentified metabolites which is automatically being stored in the Cassandra database (Fig. 1, step 6). The workflows currently provided in MetHoS target MS1 and MS2 level data and support identification by exact mass search as well as spectral matching. Figure 2 gives an example of two of these workflows which cover all steps required to analyze metabolomics data: conversion of mass spectra in an appropriate format, the quantification and normalization (Additional file 4: Table S1), the control of ionization mode, corrections of retention time distortions (Additional file 5: Table S2 and Additional file 6: Table S3), the identification of metabolites and the storage of metabolite measurements into the Cassandra database. For the quantification of metabolites both workflows rely on the software tool FeatureFinderMetabo [36] of OpenMS. Concerning the identification of metabolites the first workflow uses the AccurateMassSearch algorithm, which identifies metabolite features by comparing their exact mass to databases like HMDB [37], MassBank [38] and MoNA:Fiehn [39], while the second workflow uses the MetaboliteSpectralMatcher [40], which identifies small molecules from tandem MS spectra using a spectral library such as MassBank.

### Distributed storage

The main advantage of a NoSQL database over an SQL database is horizontal scalability and distributed storage, giving the possibility to store any amount of data, just limited by the number of utilized storage nodes. Apache Cassandra is such a NoSQL distributed database. Its masterless architecture appoints it unique among other NoSQL databases as it ensures high availability of data at all times. In combination with adjusting the replication factor to three in our Cassandra model, we are able to provide a no single point of failure model.

**Fig. 3:**
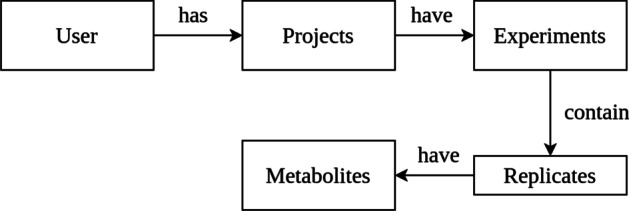
This figure shows the conceptual data model of the Cassandra database

Cassandra-specific, our data model (Fig. 3) was modeled around queries, with the *experiment* column family being the most important where the processing results of millions of metabolomic features are stored with the KNIME workflows. Furthermore, with an appropriate partitioning within Cassandra, it is made sure that data is equally distributed and stored efficiently.

### Distributed statistical analysis

Apache Spark is a fast, distributed in-memory large-scale data processing engine providing powerful Machine Learning algorithms, with their own library, which perform statistical tests in a distributed manner. MetHoS uses the Apache Spark Machine Learning library (Spark MLlib) in combination with the recently developed data structure called *Dataset*, which provides the convenience of an RDD, less memory consumption and automatic optimization. For every statistical test of our application, Apache Spark creates *jobs* which are comprised from *tasks* that are distributed from the Spark master and executed from the Spark executors on data *partitions*. Each executor of a node has been assigned a number of cores and the more cores can be used the more tasks can be performed in parallel.

The statistical methods for analysis available in MetHoS are:

- Basic statistics (mean and standard deviation)
- Metabolite filtering (mean and standard deviation between two defined groups of experiments)
- Pearson correlation
- Spearman correlation
- Principal component analysis (PCA)
- Clustering (K-means, Bisecting k-means)

In our analyses, we provide a set of choices depending on the desired depth of analysis. Specific metabolites can be selected to set the focus of the analysis. If required, handling of missing values can be incorporated. In the default settings, analyses are conducted on any common metabolite that exists in every selected experiment. Respectively, for the replicate level, the list of the metabolites that exist at least once in every replicate of the selected experiments will be selected for analysis. Missing values strategies include replacement with zero, mean or median. Results of an analysis, e.g. basic statistics such as mean and standard deviation values of all or common metabolites in experiments can be exported in .csv format for further analysis and observation.

## Results

The capabilities of MetHoS are presented with more detail in the following evaluation and a use case in which we performed a large-scale processing, storage and statistical analysis in thousands of experiments downloaded from the MetaboLights database belonging to 38 different studies [36, 41–73].

### Evaluation of scalability

**Fig. 4:**
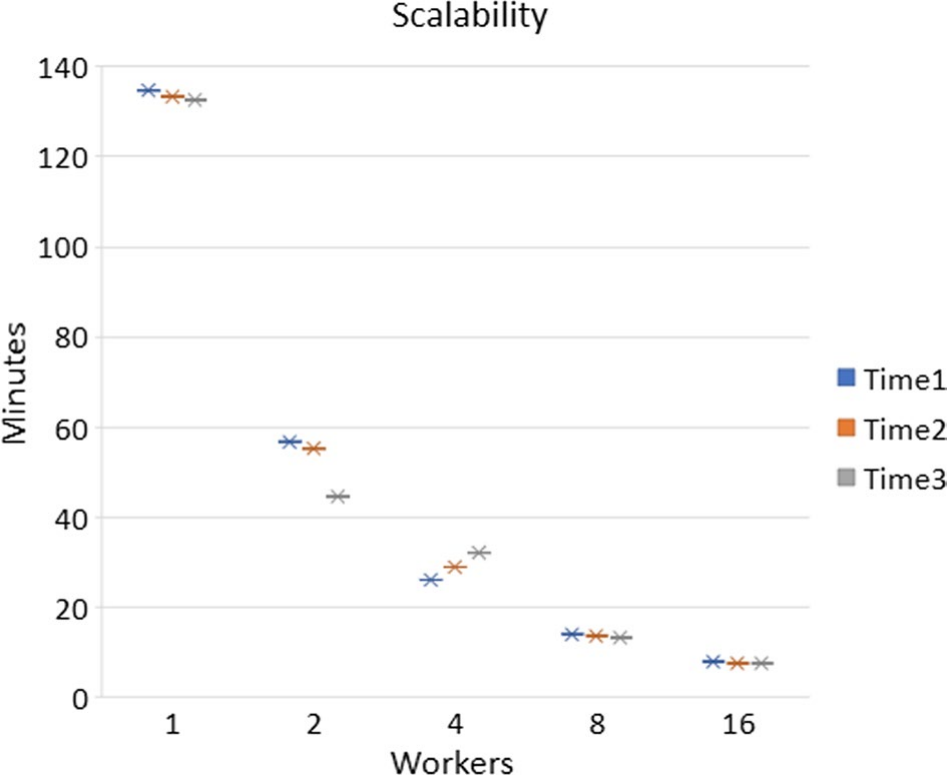
This figure shows the scalability of MetHoS with 1, 2, 4, 8 and 16 Spark workers compare to the time it takes to process 200 experiments. The processing was performed 3 times on the same 200 experiments for every number of workers

Processing thousands of experiments in a linear manner with the traditional methods would take several days or weeks depending on the complexity and size of the files. In order to prove the efficiency of parallel processing and the horizontal scalability of MetHoS, we processed 200 experiments originating from a study of MetaboLights repository, under the study identifier MTBLS28, with a variable number of Spark workers (Fig. 4). The results indicated that the more worker nodes are added in the cluster, the faster the processing is completed.

### Use case data

In our comprehensive use case, we extended to all the samples of studies from 2012 to 2020 which were downloaded and grouped in experiments, after excluding problematic and corrupted files. They originate from mass spectrometry experiments of the human organism which contain .mzData, .mzML and .mzXML files (Additional file 7: Table S4). Uploading the experiments in MetHoS resulted in 4827 experiments occupying approximately 1.1 Tb of disk space in the Openstack object storage space.

### Processing

**Fig. 5:**
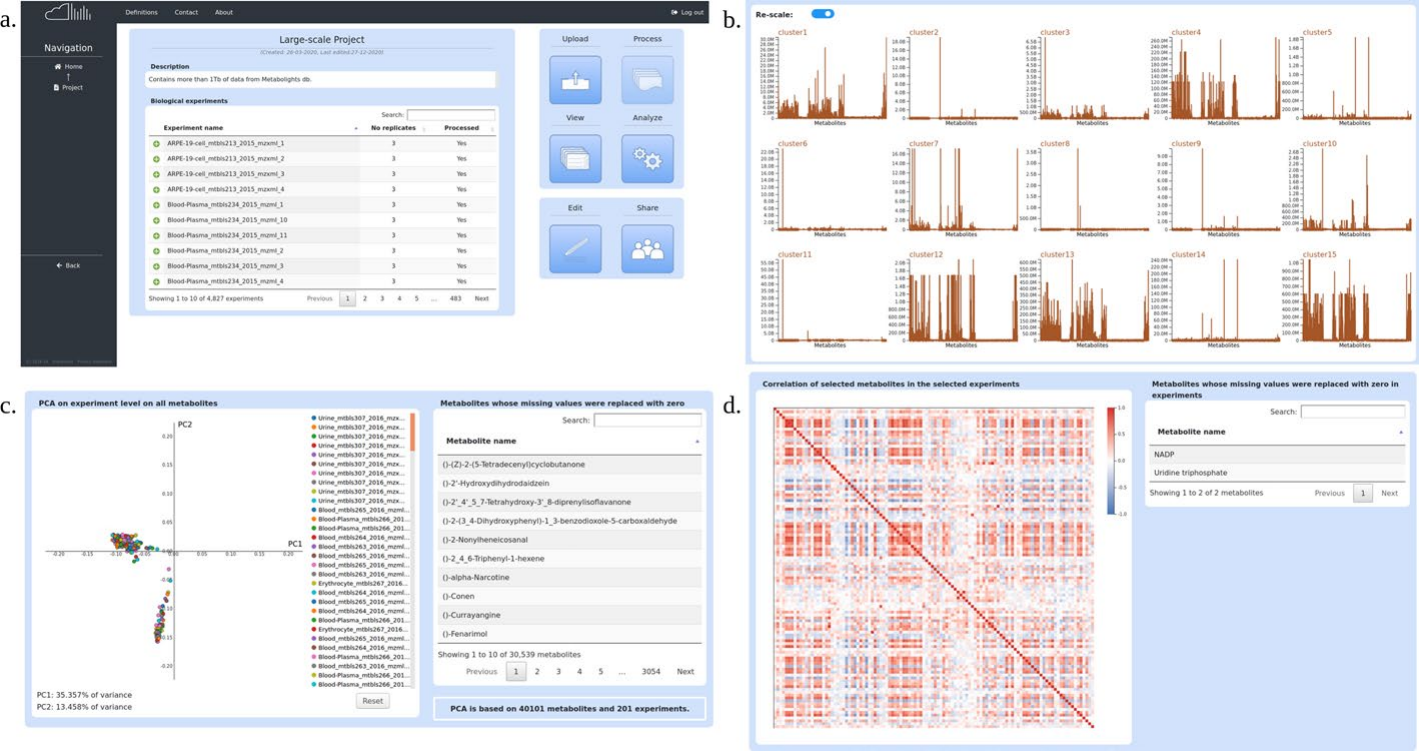
**a** Web interface of a project. **b** K-means clustering on all 4827 experiments (re-scaled). **c** PCA analysis of 144 experiments originating from whole blood, blood plasma and erythrocyte samples and 57 experiments originating from urine samples. **d** Pearson Correlation of 90 experiments on 112 compounds

The processing with MetHoS lasted approximately 12 hours and ended up quantifying and identifying more than 2 billion metabolite features. The results were automatically stored in MetHoS (Fig. 5a).

### Analysis results

For our analysis, we performed a k-means clustering in all 4.827 experiments using 15 clusters, with all metabolites, on experiment level and replacing missing values with the mean (Fig. 5b, Additional file 1: Fig. S1). Results show that two big clusters were formed, clusters 1 and 14 (Additional file 8: Table S5, Additional file 9: Table S6). Although there are similarities between several urine samples and blood or blood plasma or blood serum samples, the majority of samples in cluster 1 belong to blood samples and for cluster 14 to urine samples. Clusters 3, 6, 7, 8, 11, 12, 13 and 15 consist exclusively of urine samples, while clusters 2 and 9 only of solvent samples. Moreover, cluster 4 consists only of blood serum samples and cluster 10 only of blood plasma samples. Samples originating from lung, feces, renal tubule or cerebrospinal fluid have been split almost equally in clusters 1 and 14 indicating the formation of two distinct groups possibly originating from two different conditions in each group of experiments. Furthermore, all samples originating from THP-1-cell and Breath have been clustered together in cluster 1 and all samples from MCF-10A-cell and umbilical vein endothelial cell line in cluster 14. Finally, cluster 5 suggests similarities between some solvent and urine samples.

We selected 201 experiments (Additional file 7: Table S4) of blood and urine and performed a Principal Component Analysis on experiment level, selecting all metabolites, replacing missing values with zero and using the z-score normalization. The analysis ended up differentiating the urine samples from the blood samples successfully in an interactive PCA plot (Fig. 5c). It is shown that the intensities of 40.101 metabolites from 201 experiments took part in the calculation of the PCA, while metabolites that were not present in all the experiments are depicted in the table next to the PCA plot.

Afterwards, 90 experiments (Additional file 7: Table S4) were selected, containing blood samples from 30 individuals, 15 young and 15 elderly (30 whole blood, 30 erythrocyte and 30 blood plasma samples). Pearson Correlation was implemented on 112 compounds (Additional file 10: Table S7) that according to literature [47], show age-related increases or decreases while replacing the missing values with zero (Fig. 5d).

The results indicated that Fructose 6-phosphate and Glucose 6-phospate are highly positively correlated. The same stands for 2-Phosphoglyceric acid and 2,3-Diphosphoglyceric acid which have a very strong correlation. Closely correlated are also Nicotinamide Adenine Dinucleotide (NAD) and Nicotinamide Adenine Dinucleotide Phosphate (NADP). Last but not least, there were missing values of NADP and Uridine Triphosphate which indicates that they were not present in all 90 experiments.

Thereafter, we selected 45 of the 90 aforementioned experiments that contain samples originating from the 15 young individuals and performed a k-means clustering with 10 clusters, on metabolite level and replacing missing values with zero. The same was implemented for the rest 45 experiments of the 15 elder individuals (Additional file 2: Fig. S2, Additional file 3: Fig. S3).

The results suggest that metabolites like Leucine and Isoleucine, which may play a distinct role in supporting skeletal muscle activity, are clustered together in both cases (Additional file 11: Table S8, Additional file 12: Table S9). Ergothioneine is clustered alone in both cases showing more fluctuations in the elder individuals. In both young and elder clusters, Adenosine Diphosphate (ADP) and NAD are clustered together while Adenosine Triphosphate (ATP) is clustered separately in both cases. L-Acetylcarnitine is clustered separately for the young people while for the elder people it is clustered together with metabolites that are involved in the glucose metabolism (2-phosphoglyceric acid, 3-phosphoglyceric acid, Guanosine Triphosphate (GTP), NADP and Uridine Diphosphate Glucose) and shows a decrease of its abundance in the elder people.

### Cluster setup

MetHoS is using Apache Spark 3.0.1 in standalone mode and currenctly uses 16 worker nodes and 1 master node. Each Spark worker has one executor with two cores making it possible to parallelize two tasks per worker or 32 tasks in total. We provide 23 Gb of RAM to every executor and 115 Gb to the master node. Spark is able to access the Cassandra database through the Spark-Cassandra-Connector 3.0.0 while it is also authorized to access Openstack Object Storage for downloading the experiments to be processed every time.

In the same 16 computer nodes, we have also installed KNIME providing it with 2 Gb of RAM. Spark downloads an experiment locally on the node and activates KNIME so that it can be processed on the same node. Consequently, a number of 32 experiments can be processed in parallel on the cluster. For our Cassandra setup, we are using the same 16 nodes used as Spark workers.

## Conclusions

Here we introduced MetHoS as a flexible and easy-to-use web-based platform, based on big data frameworks, that provides automated processing, distributed storage and distributed analysis in short processing and analysis times (Additional file 13). Our aim was to provide users a bioinformatics platform for the efficient and user-friendly handling of experimental data originating from different metabolomics studies allowing in that way the integration of metabolomics data. MetHoS is built on Apache Spark to enable metabolomics data processing and analysis using KNIME and SparkML but also to constitute the basis for future analysis functionalities. We evaluated the scalability of the platform using a variable number of Spark workers and demonstrated its capabilities by handling 1.1 Tb of data which were processed in only 12 h ending up to more than 2 billion metabolite features. MetHoS allows for automated processing of large numbers of chromatographic datasets in terms of untargeted metabolite profiling, quantification and de novo identification and by that reaches a time-efficient and target-oriented integration and interpretation of metabolomics data.

## Supplementary Information

**Additional file 1: Fig. S1.** K-means clustering on all 4827 experiments (not re-scaled).

**Additional file 2: Fig. S2.** K-means clustering of 45 experiments originating of young individuals on 112 compounds, on metabolite level and replacing missing values with zero.

**Additional file 3: Fig. S3.** K-means clustering of 45 experiments originating of elder individuals on 112 compounds, on metabolite level and replacing missing values with zero.

**Additional file 4: Table S1.** List of the parameters and their values that are used in the KNIME workflow for assembling the metabolite features.

**Additional file 5: Table S2.** List of the parameters and their values that are used in the KNIME workflow for the map alignment (correcting retention time distortions between maps).

**Additional file 6: Table S3.** List of the parameters and their values that are used in the KNIME workflow for grouping corresponding features from multiple maps.

**Additional file 7: Table S4.** The study identifiers from 38 studies of the MetaboLights repository that were used for processing and analysis.

**Additional file 8: Table S5.** List of clusters and number of experiments in each one.

**Additional file 9: Table S6.** List of clusters and number of experiments in each one.

**Additional file 10: Table S7.** List of the metabolites as they appear in Pearson Correlation heatmap starting from the top left corner.

**Additional file 11: Table S8.** Clusters of the metabolites of 45 experiments originating of 15 young individuals.

**Additional file 12: Table S9.** Clusters of the metabolites of 45 experiments originating of 15 elder individuals.

**Additional file 13.** A walk through MetHoS.

## Data Availability

The datasets used and analysed during the current study are publicly available in the MetaboLights repository at https://www.ebi.ac.uk/metabolights/ and their study identifiers can be found in the Additional file 7: Table S4. Source code is available at https://gitlab.ub.uni-bielefeld.de. Project name: MetHoS; Project home page: https://methos.cebitec.uni-bielefeld.de/; Operating system(s): Platform independent; Programming language: Java, Javascript, HTML, CQL; Other requirements: Apache Spark, Apache Cassandra, Knime, Spring Framework, OpenStack; License: GPLv3; Any restrictions to use by non-academics: None.

## Abbreviations

MS: Mass spectrometry
GC: Gas chromatography
LC: Liquid chromatography
KNIME: Konstanz Information Miner
MLlib: Machine Learning library
PCA: Principal component analysis
NAD: Nicotinamide Adenine Dinucleotide
NADP: Nicotinamide Adenine Dinucleotide Phosphate
ADP: Adenosine Diphosphate
ATP: Adenosine Triphosphate
GTP: Guanosine Triphosphate
