# Supplementary figures and images for: MetHoS: a platform for large-scale processing, storage and analysis of metabolomics data
Source: BMC Bioinformatics. 2022 Jul 8;23:267. doi: 10.1186/s12859-022-04793-w (PMC9270834; doi:10.1186/s12859-022-04793-w)

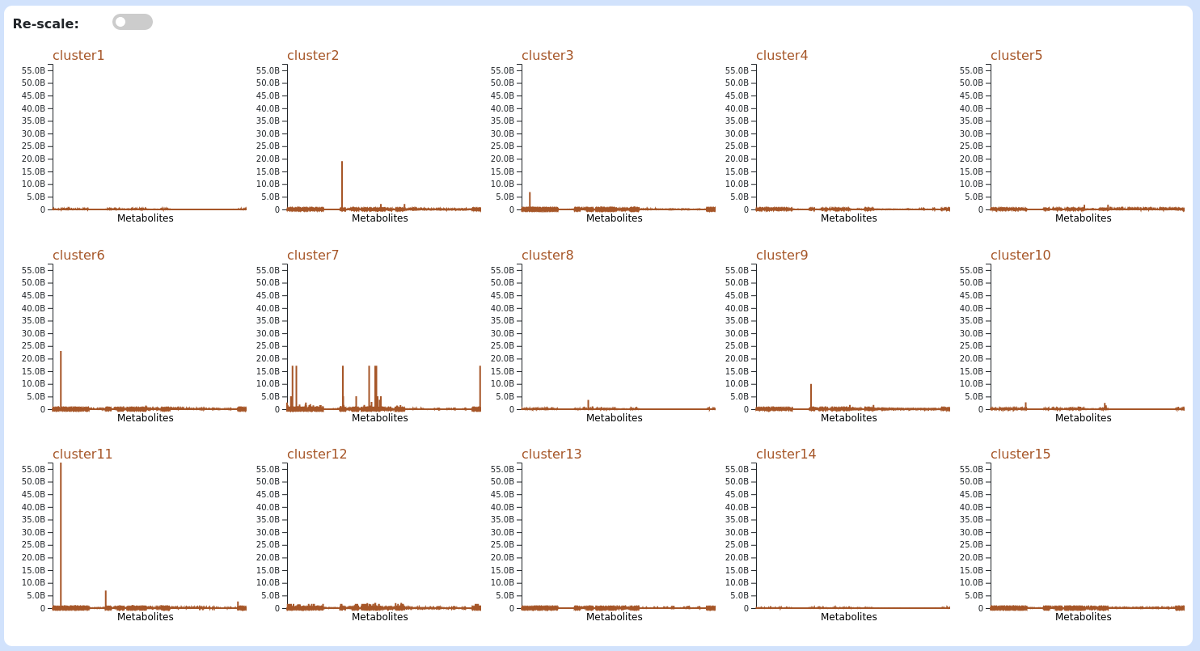

Supplement: Supplementary file 1 — Additional file 1: Fig. S1. K-means clustering on all 4827 experiments (not re-scaled). [file 12859_2022_4793_MOESM1_ESM.png]

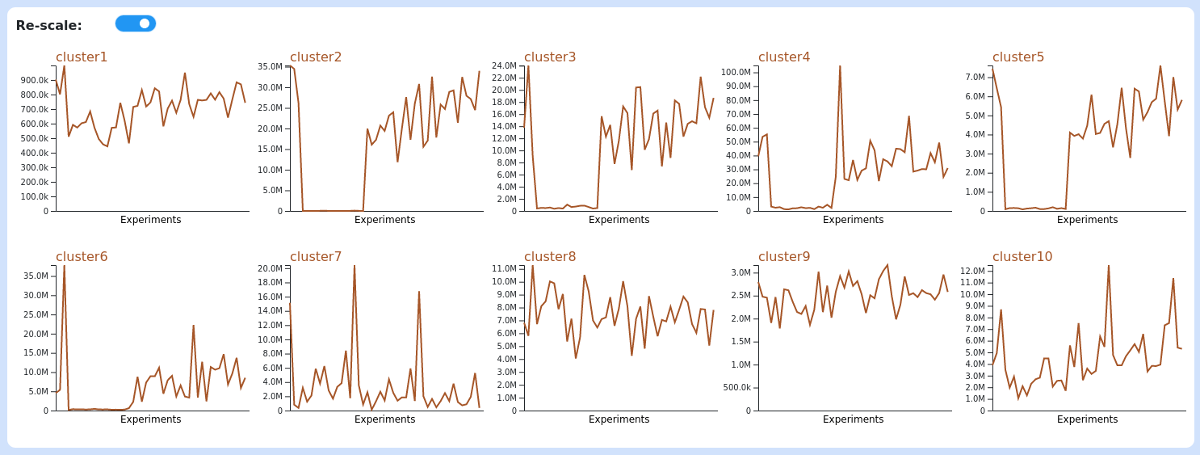

Supplement: Supplementary file 2 — Additional file 2: Fig. S2. K-means clustering of 45 experiments originating of young individuals on 112 compounds, on metabolite level and replacing missing values with zero. [file 12859_2022_4793_MOESM2_ESM.png]

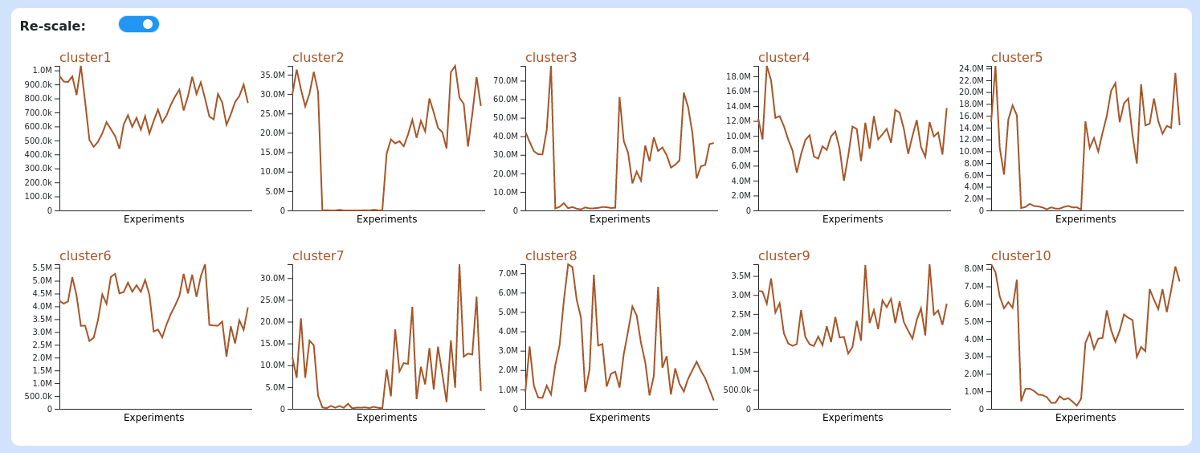

Supplement: Supplementary file 3 — Additional file 3: Fig. S3. K-means clustering of 45 experiments originating of elder individuals on 112 compounds, on metabolite level and replacing missing values with zero. [file 12859_2022_4793_MOESM3_ESM.png]
