# Supplementary material for: MetHoS: a platform for large-scale processing, storage and analysis of metabolomics data
Source: BMC Bioinformatics. 2022 Jul 8;23:267. doi: 10.1186/s12859-022-04793-w (PMC9270834; doi:10.1186/s12859-022-04793-w)
Supplement: Supplementary file 4 — Additional file 4: Table S1. List of the parameters and their values that are used in the KNIME workflow for assembling the metabolite features. [file 12859_2022_4793_MOESM4_ESM.pdf]

Table S1: List of the parameters and their values that are used currently in the KNIME workflows for assembling the metabolite features.

**Workflow** / **Parameter**	**Lenient**	**Default/MS2 Spectral Matching**	**Strict**
noise_threshold_int	8	10	12
chrom_peak_snr	2	3	3
chrom_fwhm	10	8	3
trace_termination_criterion	sample_rate	sample_rate	sample_rate
mtd: min_trace_length	2	3	3
mtd: max_trace_length	-1	-1	400
mtd: mass_error	12	10	8
width_filtering	off	off	auto
report_convex_hulls	true	true	true
