# Supplementary material for: MetHoS: a platform for large-scale processing, storage and analysis of metabolomics data
Source: BMC Bioinformatics. 2022 Jul 8;23:267. doi: 10.1186/s12859-022-04793-w (PMC9270834; doi:10.1186/s12859-022-04793-w)
Supplement: Supplementary file 5 — Additional file 5: Table S2. List of the parameters and their values that are used in the KNIME workflow for the map alignment (correcting retention time distortions between maps). [file 12859_2022_4793_MOESM5_ESM.pdf]

Table S2: List of the parameters and their values that are used currently in the KNIME workflows for the map alignment(correcting retention time distortions between maps).

Workflow / Parameter	**Lenient**	**Default/MS2 Spectral Matching**	**Strict**
max_num_peaks_considered	-1	-1	-1
mz_pair_max_distance	0.005	0.005	0.005
num_used_points	10000	10000	10000
distance_RT → max_difference	30	20	10
distance_MZ → max_difference	20	20	20
distance_MZ → unit	ppm	ppm	ppm
