# Supplementary material for: MetHoS: a platform for large-scale processing, storage and analysis of metabolomics data
Source: BMC Bioinformatics. 2022 Jul 8;23:267. doi: 10.1186/s12859-022-04793-w (PMC9270834; doi:10.1186/s12859-022-04793-w)
Supplement: Supplementary file 6 — Additional file 6: Table S3. List of the parameters and their values that are used in the KNIME workflow for grouping corresponding features from multiple maps. [file 12859_2022_4793_MOESM6_ESM.pdf]

Table S3: List of the parameters and their values that are used in the KNIME workflows for grouping corresponding features from multiple maps.

**Parameter**	**Value**
distance_RT → max_difference	40
distance_MZ → max_difference	20
distance_MZ → unit	ppm
