# Supplementary material for: MetHoS: a platform for large-scale processing, storage and analysis of metabolomics data
Source: BMC Bioinformatics. 2022 Jul 8;23:267. doi: 10.1186/s12859-022-04793-w (PMC9270834; doi:10.1186/s12859-022-04793-w)
Supplement: Supplementary file 7 — Additional file 7: Table S4. The study identifiers from 38 studies of the MetaboLights repository that were used for processing and analysis. [file 12859_2022_4793_MOESM7_ESM.pdf]

Table S4: The study identifiers from the 38 studies of the MetaboLights repository that were used for processing and analysis.

**Study identifier**	**Experiments chosen for PCA**	**Experiments chosen for Pearson**
MTBLS28		
MTBLS87		
MTBLS88		
MTBLS103		
MTBLS127		
MTBLS140		
MTBLS198		
MTBLS213		
MTBLS233		
MTBLS234		
MTBLS263	x	
MTBLS264	x	
MTBLS265	x	x
MTBLS266	x	x
MTBLS267	x	x
MTBLS277		
MTBLS307	x	
MTBLS315		
MTBLS321		
MTBLS354		
MTBLS401		
MTBLS404		
MTBLS413		
MTBLS414		
MTBLS417		
MTBLS423		
MTBLS449		
MTBLS473		
MTBLS476		
MTBLS549		
MTBLS558		
MTBLS620		
MTBLS721		
MTBLS729		
MTBLS739		
MTBLS749		
MTBLS842		
MTBLS916		
