# Supplementary material for: MetHoS: a platform for large-scale processing, storage and analysis of metabolomics data
Source: BMC Bioinformatics. 2022 Jul 8;23:267. doi: 10.1186/s12859-022-04793-w (PMC9270834; doi:10.1186/s12859-022-04793-w)
Supplement: Supplementary file 8 — Additional file 8: Table S5. List of clusters and number of experiments in each one. [file 12859_2022_4793_MOESM8_ESM.pdf]

Table S5: List of clusters and number of experiments in each one.

**Cluster**	**No experiments**
cluster1	1958
cluster2	13
cluster3	7
cluster4	137
cluster5	137
cluster6	2
cluster7	1
cluster8	35
cluster9	21
cluster10	19
cluster11	1
cluster12	1
cluster13	37
cluster14	2451
cluster15	7
