# Supplementary material for: MetHoS: a platform for large-scale processing, storage and analysis of metabolomics data
Source: BMC Bioinformatics. 2022 Jul 8;23:267. doi: 10.1186/s12859-022-04793-w (PMC9270834; doi:10.1186/s12859-022-04793-w)
Supplement: Supplementary file 9 — Additional file 9: Table S6. List of clusters and number of experiments in each one. [file 12859_2022_4793_MOESM9_ESM.pdf]

Table S6: List of clusters and number of experiments in each one.

Cluster	No experiments
**CLUSTER 1**	
ARPE-19-cell	2
Blood-Plasma	330
Blood-Serum	225
Blood	142
THP-1-cell	28
Urine	649
Beath	38
Renal-Tubule	62
Lung	130
Erythrocyte	28
Feces	18
C2C12	9
Cerebrospinal-fluid	207
Solvent	88
Pure Substance	2
**CLUSTER 2**	
Solvent	13
**CLUSTER 3**	
Urine	7
**CLUSTER 4**	
Blood-Serum	137
**CLUSTER 5**	
Solvent	51
Urine	86
**CLUSTER 6**	
Urine	2
**CLUSTER 7**	
Urine	1
**CLUSTER 8**	
Urine	35
**CLUSTER 9**	
Solvent	21
**CLUSTER 10**	
Blood-Plasma	19
**CLUSTER 11**	
Urine	1
**CLUSTER 12**	
Urine	1
**CLUSTER 13**	
Urine	37

Cluster	No experiments
**CLUSTER 14**	
ARPE-19-cell	2
Blood-Plasma	784
Blood-Serum	42
Blood	1
Cerebrospinal-fluid	47
Erythrocyte	2
Feces	10
Lung	128
MCF-10A-cell	62
Pure-Substance	50
Renal-Tubule	94
Solvent	1
Umbilical vein endothelial cell line	15
Urine	1213
**CLUSTER 15**	
Urine	7
