# Supplementary material for: MetHoS: a platform for large-scale processing, storage and analysis of metabolomics data
Source: BMC Bioinformatics. 2022 Jul 8;23:267. doi: 10.1186/s12859-022-04793-w (PMC9270834; doi:10.1186/s12859-022-04793-w)
Supplement: Supplementary file 10 — Additional file 10: Table S7. List of the metabolites as they appear in Pearson Correlation heatmap starting from the top left corner. [file 12859_2022_4793_MOESM10_ESM.pdf]

Table S7: List of the metabolites as they appear in Pearson Correlation heatmap starting from the top left corner.

Metabolite name
Isovalerylcarnitine
1-Methyladenosine
1-Methylguanosine
1-Methylhistidine
2-Phosphoglyceric acid
2_3-Diphosphoglyceric acid
3-Phosphoglyceric acid
4-Guanidinobutanoic acid
4-Trimethylammoniobutanoic acid
6-Phosphogluconic acid
7a-Hydroxy-5b-cholanic acid
acetylcarnosine
Adenine
Adenosine
Adenosine monophosphate
Adenosine triphosphate
ADP
Argininosuccinic acid
Beta-Glycerophosphoric acid
Betaine
Butyrylcarnitine
Caffeine
carnosine
CDP-Ethanolamine
Chenodeoxycholic acid
cis-Aconitic acid
Citicoline
Citramalic acid
Citric acid
citrulline
creatine
creatinine
Cytidine
Cytidine triphosphate
D-glucose
D-Glyceraldehyde 3-phosphate
D-Sedoheptulose 7-phosphate
Decanoylcarnitine
Dimethyl-L-arginine
Dodecanoylcarnitine

**Metabolite name**
Ergothioneine
Fructose 6-phosphate
GDP-glucose
Gluconic acid
Glucosamine
Glucose 6-phosphate
Glutaric acid
Glyceric acid
Glycerophosphocholine
Guanosine diphosphate
Guanosine monophosphate
Guanosine triphosphate
Hexanoylcarnitine
Hippuric acid
Hypoxanthine
Indoxyl sulfate
L-Acetylcarnitine
L-Arginine
L-Asparagine
L-Aspartic acid
L-Carnitine
L-Glutamic acid
L-Glutamine
L-Histidine
L-isoleucine
L-Kynurenine
L-leucine
L-Lysine
L-Methionine
L-Octanoylcarnitine
L-Phenylalanine
L-Proline
L-Serine
L-Threonine
L-Tryptophan
L-Tyrosine
L-Valine
Malic acid
myoInositol
N(6)-Methyllysine
N-a-Acetyl-L-arginine
N-Acetyl-D-glucosamine
N-Acetyl-L-aspartic acid

**Metabolite name**
N-Acetylglutamic acid
N-Acetylornithine
N2_N2-Dimethylguanosine
N6-acetyl-L-lysine
N6_N6_N6-Trimethyl-L-lysine
NAD
NADP
Ne_Ne dimethyllysine
Niacinamide
ophthalmic acid
ornithine
Oxoglutaric acid
Pantothenic acid
Phosphocreatine
Propionylcarnitine
Quinolinic acid
S-Adenosylhomocysteine
S-Adenosylmethionine
Succinic acid
taurine
Tetradecanoylcarnitine
Uric acid
uridine
Uridine 5'-monophosphate
Uridine diphosphate glucose
Uridine diphosphate glucuronic acid
Uridine diphosphate-N-acetylglucosamine
Uridine triphosphate
xanthine
