# Supplementary material for: MetHoS: a platform for large-scale processing, storage and analysis of metabolomics data
Source: BMC Bioinformatics. 2022 Jul 8;23:267. doi: 10.1186/s12859-022-04793-w (PMC9270834; doi:10.1186/s12859-022-04793-w)
Supplement: Supplementary file 12 — Additional file 12: Table S9. Clusters of the metabolites of 45 experiments originating of 15 elder individuals. [file 12859_2022_4793_MOESM12_ESM.pdf]

Table S9: Clusters of the metabolites of 45 experiments originating of 15 elder individuals.

**Metabolite name**
**CLUSTER 1**
1-Methyladenosine
1-Methylguanosine
1-Methylhistidine
4-Guanidinobutanoic acid
4-Trimethylammoniobutanoic acid
6-Phosphogluconic acid
7a-Hydroxy-5b-cholanic acid
Acetylcarnosine
Adenine
Argininosuccinic acid
Beta-Glycerophosphoric acid
Butyrylcarnitine
Carnosine
CDP-Ethanolamine
Chenodeoxycholic acid
cis-Aconitic acid
Citicoline
Citramalic acid
Citric acid
Cytidine
Cytidine triphosphate
D-Glyceraldehyde 3-phosphate
D-Sedoheptulose 7-phosphate
Dimethyl-L-arginine
Dodecanoylcarnitine
Fructose 6-phosphate
GDP-glucose
Gluconic acid
Glucosamine
Glucose 6-phosphate
Glutaric acid
Glyceric acid
Guanosine diphosphate
Guanosine monophosphate
Hexanoylcarnitine
Hippuric acid
Hypoxanthine
Isovalerylcarnitine
L-Asparagine

**Metabolite name**
L-Aspartic acid
L-Histidine
L-Isoleucine
L-Kynurenine
L-Leucine
L-Lysine
L-Octanoylcarnitine
L-Proline
L-Threonine
L-Tyrosine
Malic acid
N(6)-Methyllysine
N-a-Acetyl-L-arginine
N-Acetyl-D-glucosamine
N-Acetyl-L-aspartic acid
N-Acetylglutamic acid
N-Acetylornithine
N2_N2-Dimethylguanosine
N6-Acetyl-L-lysine
Ne_Ne dimethyllysine
Niacinamide
Ophthalmic acid
Ornithine
Oxoglutaric acid
Pantothenic acid
Phosphocreatine
Quinolinic acid
S-Adenosylhomocysteine
S-Adenosylmethionine
Succinic acid
Tetradecanoylcarnitine
Uridine
Uridine 5'-monophosphate
Uridine diphosphate glucuronic acid
Uridine diphosphate-N-acetylglucosamine
Uridine triphosphate
Xanthine
**CLUSTER 2**
2_3-Diphosphoglyceric acid
**CLUSTER 3**
Adenosine triphosphate
**CLUSTER 4**
L-Carnitine

**Metabolite name**
**CLUSTER 5**
ADP
NAD
**CLUSTER 6**
D-Glucose
L-Arginine
L-Glutamine
Myoinositol
**CLUSTER 7**
Ergothioneine
**CLUSTER 8**
Decanoylcarnitine
**CLUSTER 9**
Adenosine
Adenosine monophosphate
Betaine
Caffeine
Citrulline
Creatine
Creatinine
Glycerophosphocholine
Indoxyl sulfate
L-Glutamic acid
L-Methionine
L-Phenylalanine
L-Serine
L-Tryptophan
L-Valine
N6_N6_N6-Trimethyl-L-lysine
Propionylcarnitine
Taurine
Uric acid
**CLUSTER 10**
2-Phosphoglyceric acid
3-Phosphoglyceric acid
Guanosine triphosphate
L-Acetylcarnitine
NADP
Uridine diphosphate glucose
