# Supplementary material for: MetHoS: a platform for large-scale processing, storage and analysis of metabolomics data
Source: BMC Bioinformatics. 2022 Jul 8;23:267. doi: 10.1186/s12859-022-04793-w (PMC9270834; doi:10.1186/s12859-022-04793-w)
Supplement: Supplementary file 13 — Additional file 13. A walk through MetHoS. [file 12859_2022_4793_MOESM13_ESM.pdf]

MetHoS

A walk through MetHoS

A guide through process and analysis of mass spectrometry-based metabolomics data.

In the following we would like to introduce you some of the functionality of MetHoS - a web-based platform for large-scale storage, processing and analysis of metabolomics data. This walkthrough aims to guide through the system and demonstrate how to reach analysis results from raw data. MetHoS is best displayed using Mozilla Firefox, but other browsers such as Google Chrome can also be used.

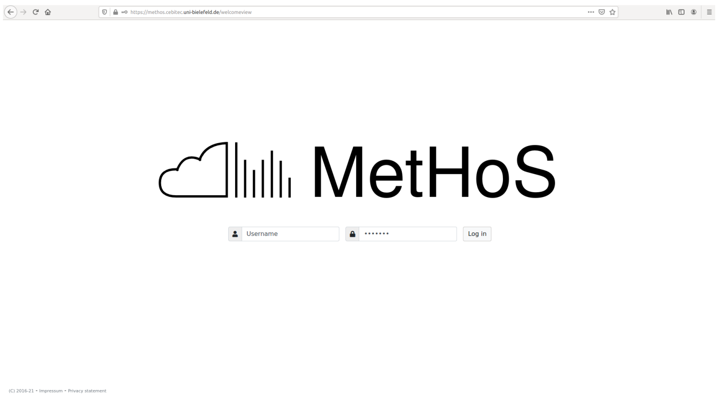

To log into the system, please first enter your username and password and click on the "Log in" button under the logo.

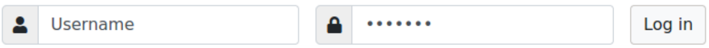

Welcome to MetHoS! On the "Home" page you will notice that you can create a project or navigate to the tabs "Definitions", "Contact" and "About" on the top left corner for more information.

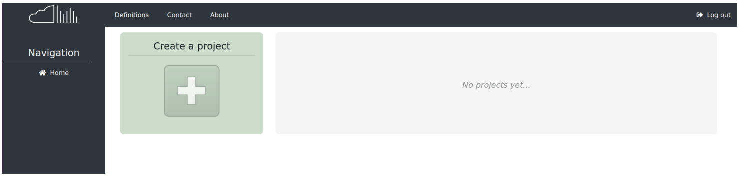

When you create a project you have to give a name, select one of the available databases and write an appropriate description. The database will be used later during the processing in the identification step.

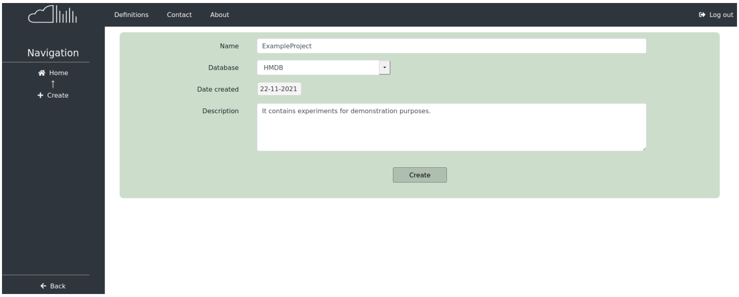

On the main "Home" page you can see all the projects you have created or have access to.

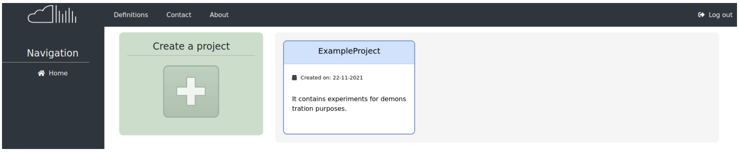

The "Definitions" and "About" tab will help you understand the concepts adopted by the tool and how it operates. There is a brief description on the functionality of MetHoS and which software is integrated in order to achieve parallel processing, distributed storage and distributed analysis.

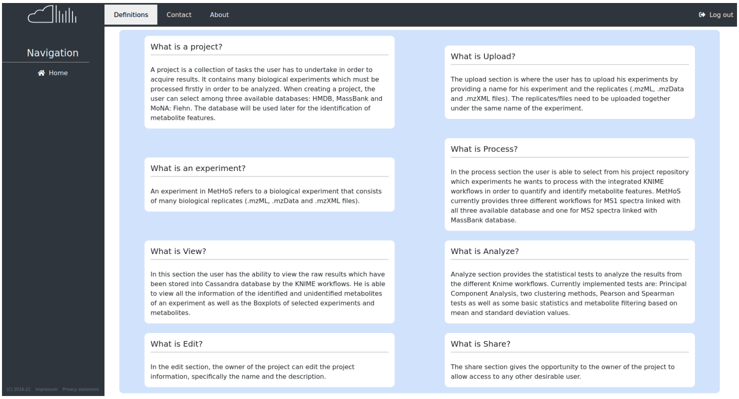

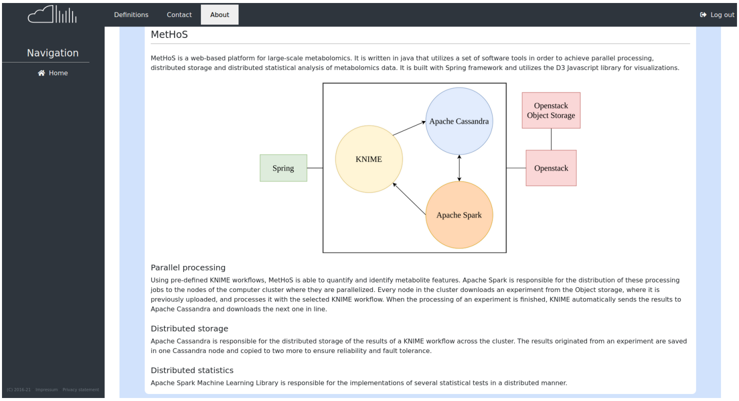

On the main project page you can see the information of the project and some available options. The creator of the project is the owner of the project and the only user who can give or remove access rights of the project with the "Share" button. Also, the owner is the only user who has the right to edit the name and description of the project with the "Edit" button.

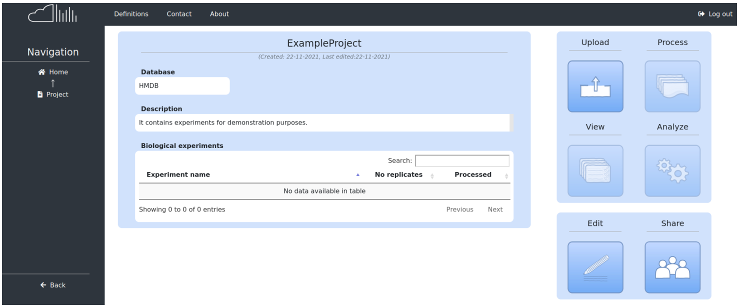

With the "Upload" button you can upload your experiments. Each experiment refers to a biological experiment that may consist of many biological replicates (files). The replicates must be grouped and uploaded together for every experiment. For our example, we have randomly selected 10 blood plasma experiments of the mtbls315 study and 10 urine experiments of the mtbls28 study of the MetaboLights database.

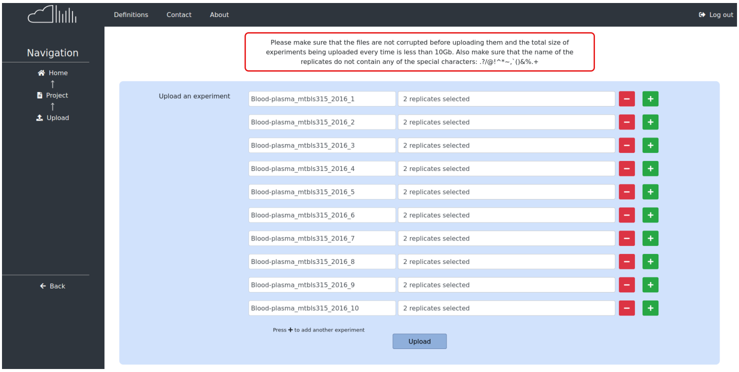

After upload finishes, you will be able to see the list of experiments you uploaded, as well as their replicates, on the main panel of the project. You can also see that as soon as you have unprocessed experiments in the project, the ”Process” button becomes available.

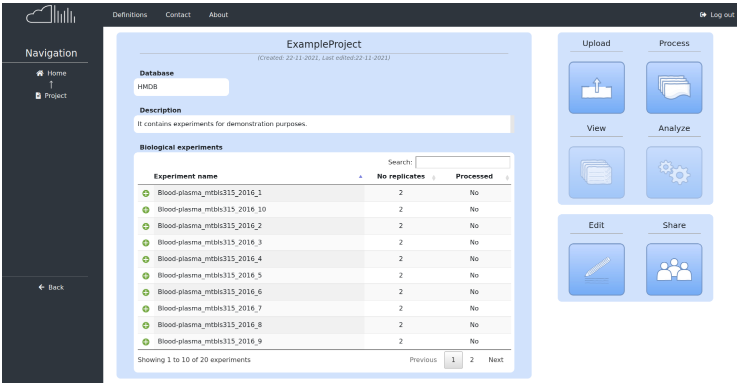

With the ”Process” button you can select the experiments you want to process (quantification and identification) and choose one of the currently available workflows.

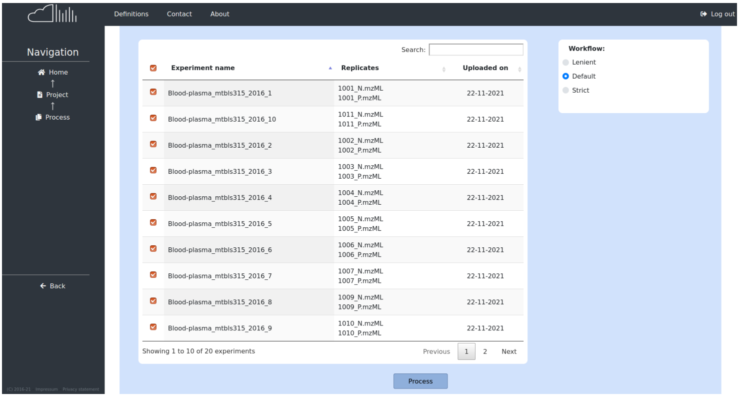

As soon as the processing is finished, the buttons "View" and "Analyze" become available. With the "View" button you are able to observe the raw data in a form of table or represent them in box plots.

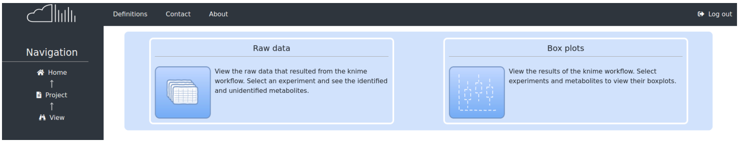

In the Box plots section you can see the distribution of metabolites in the selected experiments. A list of all the metabolites that are present at least once in at least one experiment is presented on the right. You can select any metabolite of this list and observe the boxplots with the outliers. Selecting the checkbox "Show metabolites" will present all the metabolites that are within the quartiles. In this specific example, you can see that the metabolite ()-4-Methylene-2-pyrrolidinecarboxylic acid has a bigger distribution in the blood plasma experiments than the urine experiments.

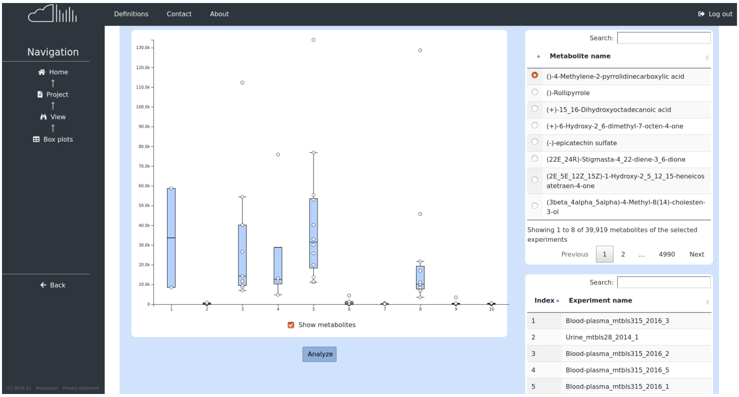

With the "Analyze" button you can perform several statistical tests such as PCA, clustering and more.

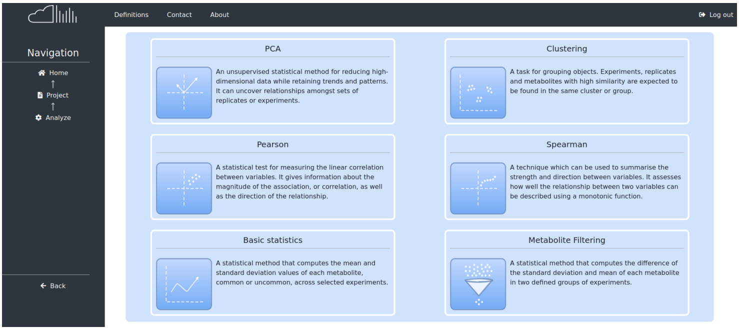

For most of the analysis tests you have the choice to select all or specific metabolites and handle missing values imputation by omitting or replacing them with the zero, mean or median values. Furthermore, you are able to define the depth of your analysis by choosing the level depending on what you want to focus on, e.g. experiments, replicates or metabolites.

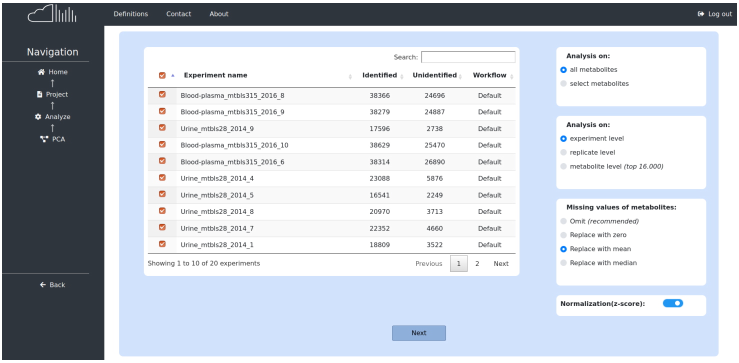

Following, you can see the results of a PCA of all 20 experiments on all metabolites in experiment level. The missing values are replaced with the mean and normalized. The metabolites that were replaced by the mean are presented as a list on the right side as well as the total number of metabolites and experiments that participated in the analysis. If you hover over the plot you will see the names of the experiments while also you can zoom in the plot or select any experiment of the list on the right side to highlight it. In this example, we can see that there are two distinct groups, one with all the blood plasma experiments and one with all the urine experiments.

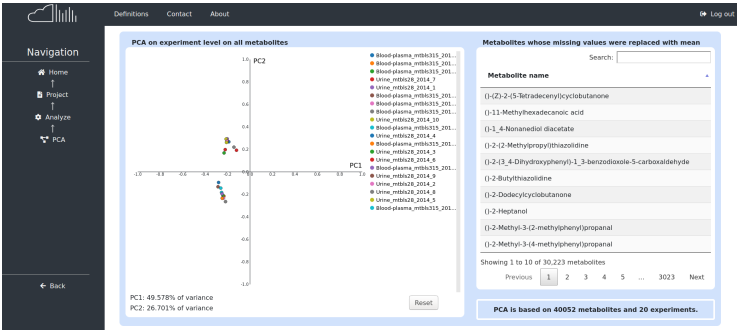

Thank you very much for your interest in MetHoS.

If you have further questions please do not hesitate to contact us.
